# No direct evidence for the presence of Nubian Levallois technology and its association with Neanderthals at Shukbah Cave

**DOI:** 10.1038/s41598-022-05072-7

**Published:** 2022-01-24

**Authors:** Emily Hallinan, Omry Barzilai, Nuno Bicho, João Cascalheira, Yuri Demidenko, Mae Goder-Goldberger, Erella Hovers, Anthony Marks, Maya Oron, Jeffrey Rose

**Affiliations:** 1 Interdisciplinary Center for Archaeology and Human Behaviour, Universidade do Algarve, 8005-139 Faro, Portugal; 2 Israel Antiquities Authority, Rockefeller Museum, 91004 Jerusalem, Israel; 3 Ferenc Rakoczi II Transcarpathian Hungarian College of Higher Education, Berehove, 902 02 Ukraine; 4 Institute of Archaeology NASU, Kyiv, 042 10 Ukraine; 5 Department of Bible, Archaeology and the Ancient Near East, Ben-Gurion University of the Negev, 84105 Beersheba, Israel; 6 Institute of Archaeology, The Hebrew University of Jerusalem, Mt. Scopus, 9190501 Jerusalem, Israel; 7 Department of Anthropology, Southern Methodist University, Dallas, TX 75275 USA; 8 Ronin Institute, Montclair, NJ 07043 USA

**Keywords:** Biological anthropology, Archaeology

**arising from**: J. Blinkhorn et al.; *Scientific Reports* 10.1038/s41598-021-82257-6 (2021).

Blinkhorn et al.^1^ present a reanalysis of fossil and lithic material from Garrod’s 1928 excavation at Shukbah Cave, identifying the presence of Nubian Levallois cores and points in direct association with a Neanderthal molar. The authors argue that this demonstrates the Nubian reduction strategy forms a part of the wider Middle Palaeolithic lithic repertoire, therefore its role as a cultural marker for *Homo sapiens* population movements is invalid.

We raise the following four major concerns: (1) we question the assumptions made by the authors about the integrity and homogeneity of the Layer D assemblage and (2) the implications of this for the association of the Neanderthal tooth with any specific component of the assemblage, (3) we challenge the authors’ attribution of lithic material to Nubian Levallois technology according to its strict definition, and (4) we argue that the comparative data presented derive from a biased sample of sites. These points critically undermine the article’s conclusion that Shukbah’s Neanderthals made Nubian cores and thus the argument that Neanderthals *might* have made Nubian technology elsewhere is unsubstantiated.

## Shukbah Layer D cannot be treated as a single unmixed assemblage

The Layer D lithic assemblage derives from a brecciated deposit ranging in thickness from 0.2 to 2.5 m, with Layer D material said to be redeposited as disturbed Layer C^2,3^. In the context of a Palaeolithic cave, a deposit this thick inevitably combines multiple occupation phases that were excavated as a single unit; thus, it is problematic to treat the assemblage as a homogeneous entity. Renewed excavations at multiple key sites in Israel have highlighted the need for caution when referring to stratigraphy described in early twentieth century excavations; for example, at Tabun Cave^4,5^ following Garrod's^6^ original excavation, and at Kebara Cave^7,8^ after Turville-Petre^9^. At both sites, thick and extensive Middle Palaeolithic strata were identified originally but subsequent investigations have shown that these units were in fact composed of many archaeological layers spanning a broad time-range. Since Garrod used a similar field methodology and parameters for defining the stratigraphic units at both Shukbah and Tabun, it is safe to regard Shukbah Layer D as similarly conflating multiple archaeological layers. Other studies of ‘old’ collections have also indicated the problems of stratigraphy from these early excavations and the consequent contextual uncertainties of the lithic assemblages^10,11^. We argue that these problems also apply to Shukbah, thereby undermining Blinkhorn et al.’s assumption that Layer D represents a single, archaeologically meaningful assemblage.

**Figure 1:**
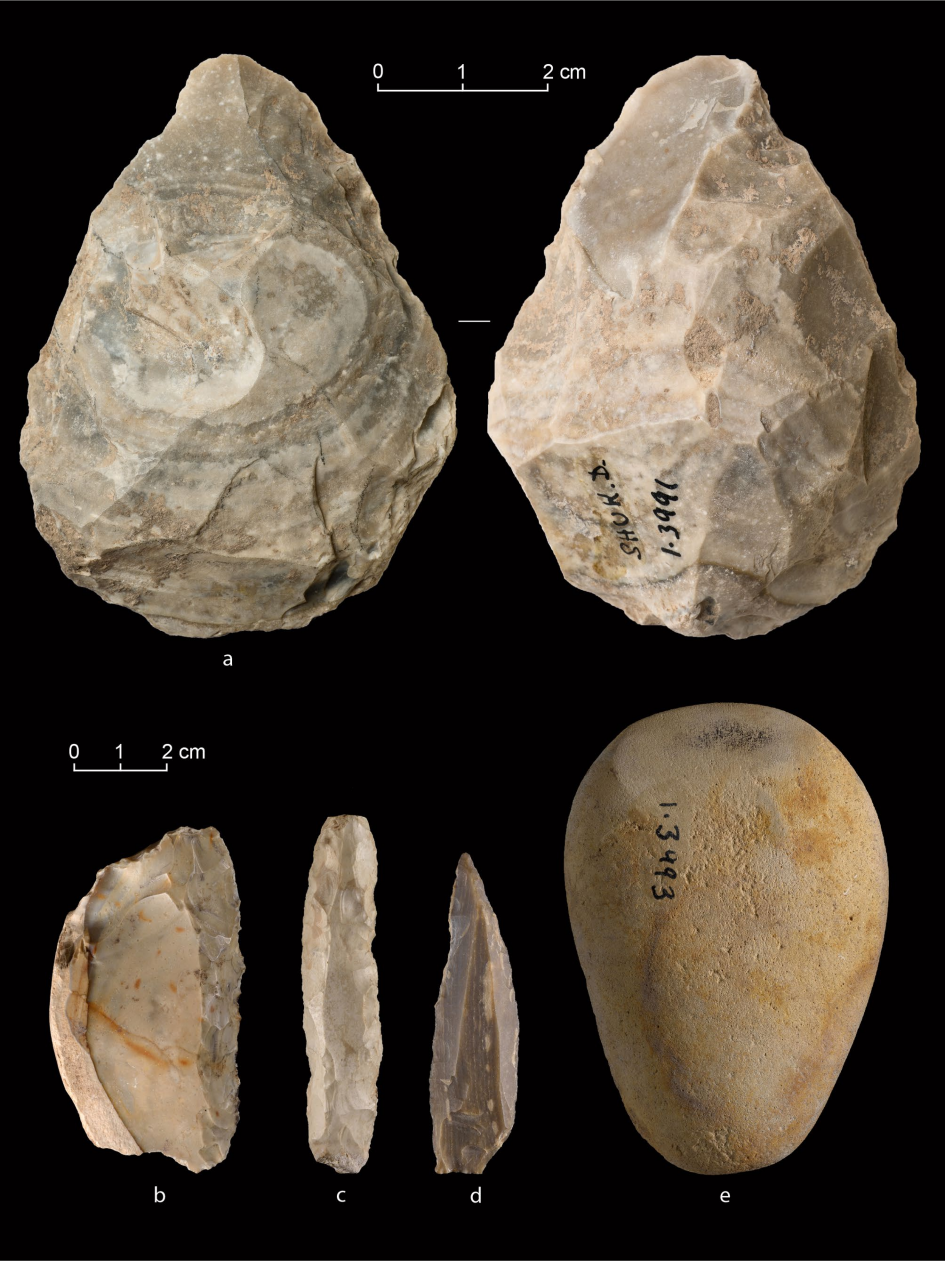
Lithic artefacts from Shukbah Layer D: (**a**) handaxe; (**b**) Acheulo-Yabrudian scraper; (**c**) retouched Aurignacian blade; (**d**) el-Wad-point; (**e**) polished axe on a pebble.

To corroborate this, we revisited the lithic collection from Shukbah D housed at the Rockefeller Museum, Jerusalem, that was not analysed by Blinkhorn et al. The Layer D collection includes 156 artefacts, of which around 75% are tools and cores (Table 1). This collection is demonstrably biased, as is the case for the analysed sample presented by Blinkhorn et al. [ref.^1^. SI7]. While the majority of the assemblage in the Rockefeller Museum collection represents the Middle Palaeolithic, artefacts from earlier and later periods are also evident (Fig. 1; Tables 2 and 3). We identified Lower (Fig. 1a-b; Table 2) and Upper Palaeolithic elements, as well as a Neolithic/Chalcolithic polished axe (Fig. 1c-e).

**Table 1:** General composition of Rockefeller Museum Shukbah Layer D sample.

Artefact type	n	%
Levallois debitage	31	19.9
Core trimming elements	3	1.9
Retouched tools	85	54.5
Cores	34	21.8
Stone pounders	3	1.9
Total	156	100.0

**Table 2:** Typology of retouched items in the Rockefeller Museum Shukbah Layer D sample.

Tool type	n	%	Period	Phase
Single side scraper	21	24.7	MP	
Double side scraper	17	20.0	MP	
Convergent side scraper	4	4.7	MP	
Scraper on massive blades	5	5.8	MP	Early MP
Hummal Point	2	2.4	MP	Early MP
Ventral side scraper	4	4.7	MP	
Ret. Levallois flake	3	3.5	MP	
Ret. Levallois blade	2	2.4	MP	
Mousterian Point	1	1.2	MP	
End scraper	3	3.5	UP, EPI?	
Burin	13	15.3	MP and UP?	
Quina scraper	1	1.2	LP	Acheulo-Yabrudian
Handaxe	2	2.4	LP	
Adze	1	1.2	Chalcolithic?	
Biface	1	1.2	Epi	Natufian?
Polished axe	1	1.2	Neolithic/Chalcolithic	
El-Wad point	1	1.2	UP	Ahmarian
Retouched blade	1	1.2	UP	Aurignacian
Notch	1	1.2	?	
Awl	1	1.2	MP	
Total	85	100.0

**Table 3:** Typology of cores in the Rockefeller Museum Shukbah Layer D sample.

Core type	n	%	Period
Levallois core	24	70.6	MP
Levallois core on flake	1	2.9	MP
Levallois preform	3	8.8	MP
Hierarchical surface core	2	5.9	MP
Blade core	2	5.9	UP?
Bladelet core	1	2.9	Epi?
Amorphous core	1	2.9	?
Total	34	100.0	

**Figure 2:**
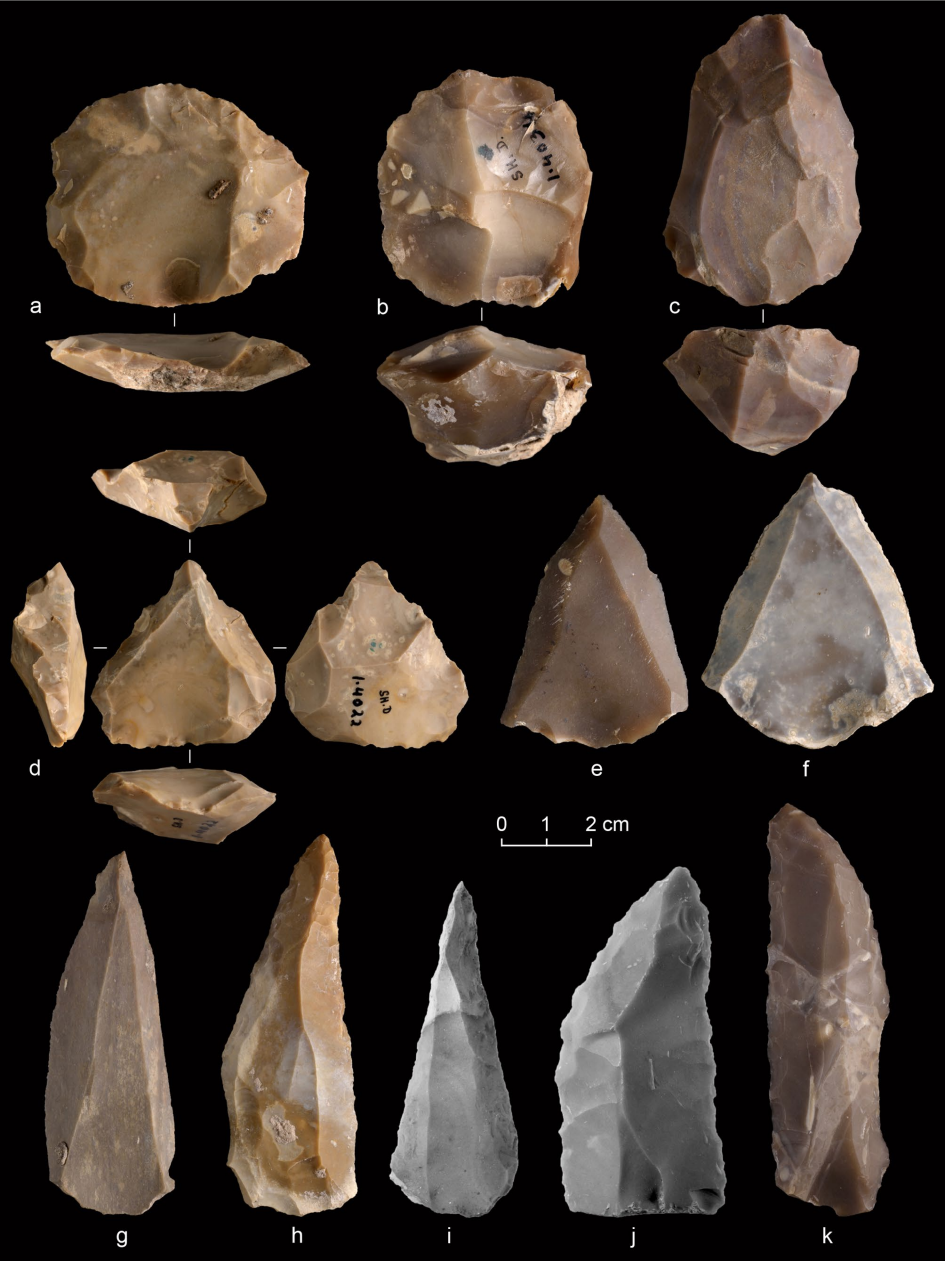
Middle Palaeolithic lithic artefacts from Shukbah Layer D: (**a**) preferential centripetally prepared Levallois core, (**b**) recurrent centripetal Levallois core; (**c**) bidirectional Levallois core; (**d**) exhausted preferential centripetally prepared Levallois core; (**e–g**) Levallois points; (**h–i**) Hummal points; (**j–k**) convergent scrapers on blades.

The Middle Palaeolithic assemblage (housed at the Rockefeller museum) is dominated by the centripetal Levallois method (Fig. 2a-b, Table 4). Bidirectional and unidirectional convergent methods are less prominent (Fig. 2c, e–g). An exhausted core bears a superficial resemblance to a Nubian core in terms of its morphology and short, steep distal ridge (42°) (Fig. 2d). Without an in-depth technological analysis, it is unclear whether this core resulted from a true Nubian reduction scheme, or is a heavily reduced centripetal core. Parsimony (see below) suggests the latter. Notably, there are several tools in the collection that were made on massive blades, including convergent scrapers, intensively retouched blades and two Hummal points (Fig. 2h-k). These are typical of the so-called ‘Tabun-D’ tradition of the early Middle Palaeolithic^12,13^. Additionally, the dominance of centripetal Levallois cores is a feature of MIS 5 assemblages^14,15^.

**Table 4:** Dorsal scar pattern (where identifiable) on Levallois artefacts.

Levallois artefact	Centripetal	Bidirectional	Convergent	n
Levallois cores	17	2	0	19
Levallois debitage	16	5	8	29

In sum, the mixed collection from Shukbah Layer D in the Rockefeller Museum calls into question the integrity of Layer D as a whole. Furthermore, since technological and typological characteristics from different chronological stages within the Middle Palaeolithic are also present, ascribing unprovenanced sub-samples of the entire assemblage to the late Middle Palaeolithic^1^ is unwarranted.

## It is unsupported to claim the “direct association” of a Neanderthal molar with specific artefact types within Layer D

Given the above, it is neither possible to define the stratigraphic component of Layer D that is associated with the Neanderthal tooth, nor to assert its association with the ‘Nubian’ artefacts in the assemblage such as they are (see below). Garrod^2^ describes that the Neanderthal molar was “found lying upon the rock at the base of the breccia, at a point where it was extremely hard throughout”, specifying that this was “the base of the hummock on the edge of the pit”^3^. Based on this description and the illustrated excavation section^3^, the molar derives from the contact between Layer D and the sediments of Layer B, giving it an insecure stratigraphic attribution. Garrod herself questioned the association of other human remains found within the Layer D breccia^2^, also described elsewhere^16^.

## No convincing data are presented to justify the identification of Nubian Levallois artefacts

Nubian Levallois technology is regarded as a distinct method of Levallois reduction; Levallois cores with distal and lateral preparation are not necessarily Nubian cores and can equally be attributed to the Levallois bidirectional and centripetal modes of core preparation^17–19^. The definition offered in Blinkhorn et al. [ref.^1^ Methods] states that “Nubian Levallois points and cores have been differentiated from other Levallois point reduction approaches by the presence of a steep medial-distal ridge produced through a combination of distal divergent or lateral removals which help to guide the preferential flake removal”. They cite Usik et al.’s^20^ suggested set of attributes against which an identification of Nubian cores can be tested and differentiated from other Levallois core types. In this definition, the reduction strategy results in: (1) a steeply angled median distal ridge < 120° and generally > 60°; (2) an opposed striking platform with angle of intersection to the exploitation surface varying from 50° to 90°; (3) a triangular or sub-triangular core shape; and (4) a faceted primary striking platform. These criteria represent the culmination of a methodological consensus from several teams working in different contextual areas^18,21–23^. These definitive criteria have been followed in numerous subsequent studies^24–27^. Blinkhorn et al.^1^ neither follow these criteria, nor present data to fully evaluate the presence of Nubian technology in Shukbah Layer D.

They do not define the steepness of the median distal ridge on Nubian cores and there are no quantitative or qualitative attributes provided to support this. They identify 16 cores as Nubian, three showing proximal and distal divergent shaping, and 13 with orthogonal or centripetal removals [ref.^1^ SI7: 20]. With the limited information provided, supported solely by illustrations of eight cores [ref.^1^ Fig. 5a-h], most of which are shown only in plan view, it is impossible to determine whether the cores considered to be ‘Nubian’ comply with the criteria outlined above and, thus, whether they can be differentiated from Levallois bidirectional or centripetal cores. The assemblage also contains 13 “other” Levallois point cores, yet no further details are given with regard to their exploitation strategy and morphological attributes.

When Blinkhorn et al. refer to the presence of 12 Nubian Levallois points [ref.^1^ Fig. 5j-n], they incorrectly presume that there is a clear definition of Nubian end-products offered elsewhere^20^. In the absence of direct refits, Usik et al. do not consider Nubian points to be distinguishable from the end-products of centripetal Levallois reduction; they observe only that “the Nubian Levallois method is based on the production of elongated Levallois points or pointed flakes”^20^. Based on extensive refitting, Van Peer^18^ describes Nubian end-products as pointed flakes produced following the central guiding ridge in the distal core part combined with a radial pattern of preparation in the proximal part. At Nazlet Khater 1 in the Nile Valley, Nubian end-products have more dorsal scars than classical Levallois end-products (i.e. from preferential centripetal Levallois cores) and tend to be longer, less elongated and thicker^28^. Given this lack of consensus, it is unclear how Blinkhorn et al.^1^ separated Nubian end-products from other Levallois end-products (points) for their statistical treatment. They identify nine ‘Nubian’ points with bidirectional dorsal scar patterns and three with a combination of distal and lateral removals [ref.^1^ SI7: 19]. However, “other Levallois points” include 49 with bidirectional scar patterns, alongside unidirectional, unidirectional convergent and centripetal removals [ref.^1^ SI Table 22]. The multivariate analyses are said to indicate that Nubian Levallois points are not distinct from the wider body of Levallois points at the site [ref.^1^ Fig. 6a, SI Fig. 2]. This is circular reasoning since the reasons for distinguishing them in the first place are not adequately justified.

In the context of the late Levantine Middle Palaeolithic record, which is characterised by a wide range of Levallois strategies^29–31^, a more detailed presentation of the data and specific attributes is required to assess the presence of Nubian technology as distinct from other Levallois methods at Shukbah. Bidirectional Levallois flaking is an integral component of almost all Middle Palaeolithic assemblages in the Levant, especially in cave sites in the Mediterranean ecozone^14,32–34^. In most places where Nubian technology is present in Arabia, the Negev desert and the Nile Valley, it is accompanied by Levallois bidirectional and centripetal modes of core preparation, while the Levallois unidirectional convergent method is almost always absent^20,22,25,26^. Therefore, given the data presented, it is more parsimonious that the Shukbah assemblage represents a continuum of bidirectional and centripetal Levallois flaking rather than a deliberate focus on Nubian Levallois reduction.

##  The multivariate analyses and comparative assemblages are not informative

Although reference is made to metric and categorial traits listed in previous studies by the authors [ref.^1^ SI7: 18], the attributes upon which the multivariate analyses are based in this paper are solely basic linear measurements and derived indices [ref.^1^ Methods]. These attributes have been shown to be highly dependent on quality and size of raw material, independent of reduction intensity^20^ but these aspects are not reported on by Blinkhorn et al.^1^. Indeed, it is doubtful that they can be, given the biased collection and outdated excavation practices.

No appropriate technological comparisons are made between different Levallois reduction strategies at Shukbah (e.g. scar directions, steepness of median distal ridges), that justify the grouping into Nubian and Other Levallois [ref.^1^ Fig. 6, SI Figs. 3 & 4]. Given that the analysed artefacts are not shown to fulfil the criteria for Nubian Levallois technology and instead represent other Levallois methods, the conclusion that “Nubian Levallois reduction strategies form part of a wider Levallois point production strategies, rather than a discrete technological approach” [ref.^1^ SI7: 20] is contained in the methodological premises and, thus, inevitable.

The choice of comparative assemblages is not well explained and sampling procedures are not justified. Problems include a reliance on very small samples (e.g. < 1% of the Rosh Ein Mor assemblage^35^) and core type totals differ from those published elsewhere with no explanation given (e.g. A5:^36^; BNS:^37^. Furthermore, they exclude the three Nubian cores from A5 at Aduma^36^, and do not include Levantine or Northeast African^38^ assemblages with Nubian technology. The observation that Nubian Levallois point cores from southern Arabia are distinct from other assemblages is unsurprising because these are the only assemblages they cite containing Nubian cores, therefore they represent a different Levallois reduction strategy [ref.^1^ Fig. 6f., SI Fig. 9]. Put simply, the multivariate analyses do no more than indicate the extent of inter-assemblage variability which is expected given the broad spatial, temporal and contextual range of assemblages compared.

## Conclusion

Just as the authors argue that “any association between *Homo sapiens* and Nubian Levallois technology remains to be demonstrated” [ref.^1^ SI3: 8], when the available data are properly evaluated, the same can also be said for its association with Neanderthals. Blinkhorn et al. do not demonstrate any association between Nubian technology and Neanderthals at Shukbah Cave. The arguments we present question the integrity of Shukbah Layer D, finding no convincing evidence for associating the Neanderthal molar with any specific artefact types within the mixed assemblage, as shown by our review of the Rockefeller Museum collection. Furthermore, the identification of so-called Nubian cores and points is insufficiently supported by the results presented and these do not explicitly demonstrate the strict criteria accepted by scholars in other recent studies, nor do they present new ones. Lastly, the comparative analyses used to contextualise Shukbah reflect biased samples that are not informative without more detailed consideration of context, raw material and other relevant technological variables.

As our knowledge of the timing and distribution of Nubian Levallois technology comes into focus, it is becoming increasingly clear that this reduction strategy occupied a continuous landscape – a contextual area spanning Northeast Africa, the Arabian Peninsula, and southern Levant – coinciding with a critical stage of modern human emergence. Blinkhorn et al.’s paper demonstrates one essential point: a unified definition of Nubian technology is fundamental to a better understanding of its significance in the processes of adaptation, cultural convergence and dispersal by modern humans in this region during the Late Pleistocene.
